# Validating a tool to measure auxiliary nurse midwife and nurse motivation in rural Nepal

**DOI:** 10.1186/s12960-015-0021-7

**Published:** 2015-05-12

**Authors:** Joanna Morrison, Neha Batura, Rita Thapa, Regina Basnyat, Jolene Skordis-Worrall

**Affiliations:** Nick Simons Institute, Box 8975, EPC 1813, Sanepa, Lalitpur Nepal; Institute for Global Health, University College London, 30 Guilford Street, London, WC1N 1EH UK

**Keywords:** Retention, Measure, Quantitative, Qualitative, South Asia

## Abstract

**Background:**

A global shortage of health workers in rural areas increases the salience of motivating and supporting existing health workers. Understandings of motivation may vary in different settings, and it is important to use measurement methods that are contextually appropriate. We identified a measurement tool, previously used in Kenya, and explored its validity and reliability to measure the motivation of auxiliary nurse midwives (ANM) and staff nurses (SN) in rural Nepal.

**Method:**

Qualitative and quantitative methods were used to assess the content validity, the construct validity, the internal consistency and the reliability of the tool. We translated the tool into Nepali and it was administered to 137 ANMs and SNs in three districts. We collected qualitative data from 78 nursing personnel and district- and central-level stakeholders using interviews and focus group discussions. We calculated motivation scores for ANMs and SNs using the quantitative data and conducted statistical tests for validity and reliability. Motivation scores were compared with qualitative data. Descriptive exploratory analysis compared mean motivation scores by ANM and SN sociodemographic characteristics.

**Results:**

The concept of self-efficacy was added to the tool before data collection. Motivation was revealed through conscientiousness. Teamwork and the exertion of extra effort were not adequately captured by the tool, but important in illustrating motivation. The statement on punctuality was problematic in quantitative analysis, and attendance was more expressive of motivation. The calculated motivation scores usually reflected ANM and SN interview data, with some variation in other stakeholder responses. The tool scored within acceptable limits in validity and reliability testing and was able to distinguish motivation of nursing personnel with different sociodemographic characteristics.

**Conclusions:**

We found that with minor modifications, the tool provided valid and internally consistent measures of motivation among ANMs and SNs in this context. We recommend the use of this tool in similar contexts, with the addition of statements about self-efficacy, teamwork and exertion of extra effort. Absenteeism should replace the punctuality statement, and statements should be worded both positively and negatively to mitigate positive response bias. Collection of qualitative data on motivation creates a more nuanced understanding of quantitative scores.

## Introduction

In low-income countries, difficulty in retaining rural health workers has increased the importance of supporting and motivating existing staff, both to maximise their productivity and encourage their retention [1]. Developing appropriate strategies and monitoring progress requires tools to measure and assess the motivation of health workers in low-income settings. Motivation measurement tools have been well developed for use in industrialised countries and usually assess personality-related characteristics and preferences for work conditions. These tools have been used to measure individual differences in values related to the utility of working hard, and the strength of intrinsic motives for performance among different employees [2]. While generic concepts of worker motivation may be relevant in all country situations, the sociocultural context may affect the importance of different determinants and the relationship between them [3]. Therefore, it is important to test and adapt tools to fit with local contexts [4].

Motivation in the workplace can be defined as “an individual’s degree of willingness to exert and maintain an effort towards organisational goals” [3]. Sources of motivation may be intrinsic, whereby an individual is motivated by an internal desire, and extrinsic, whereby rewards or incentives motivate an individual [5]. A less motivated person may perform adequately in minimally demanding conditions, but their willingness to exert extra effort into task completion depends on whether they feel there is personal value in doing so [2]. If organisational and individual goals are similar, then exerting extra effort to complete tasks benefits both the individual and the organisation, which has a positive effect on worker performance.

**Figure 1:**
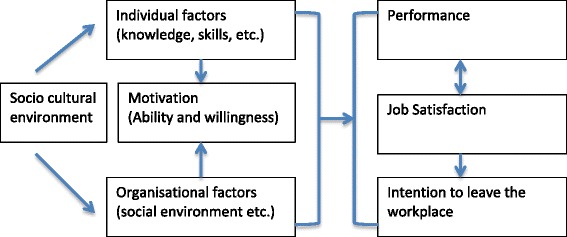
Worker motivation [6].

Contextual or environmental factors also affect motivation. If a workplace does not permit effective working, this might demotivate a health worker. If a worker does not have the knowledge, skills or experience to complete their tasks, this also adversely affects motivation. Worker motivation is thus the ability and willingness to put in the effort required to do the job, which is influenced by individual factors (such as knowledge, skills, experiences, psychological attributes) and organisational factors (such as physical and social environment, policies and practices) (Figure 1) [6]. Organisational factors are more amenable to change than individual personality tendencies, or societal and cultural values. Organisational change may also have bigger effects on motivation and could be used to promote long-term change in personal or cultural values of workers [2].

The research described in this paper is part of a larger study investigating the effect of organisational change in health facilities in rural Nepal, stimulated by national government recommendations on auxiliary nurse midwife (ANM) and staff nurse (SN) recruitment. In Nepal, maternal health indicators are improving, but the maternal mortality ratio remains high at 281 per 100 000 live births, and newborn mortality is 33 per 1000 live births. Most women deliver at home [7]. In response to these health indicators, the Government of Nepal has made long-term and short-term strategies for maternal and newborn health. The long-term strategy involves starting midwifery training and introducing a midwife cadre to Nepal [8,9]. The short-term strategy has several components: to increase access to maternal health services through establishing ‘birthing centres’—enabling existing health institutions to provide delivery care; to develop basic midwifery skills of nurse cadres through a 60-day in-service skilled birth attendant (SBA) training course [10]; and discontinuing the maternal and child health worker (MCHW) cadre, offering eligible MCHWs to take ANM training. This ANM training for MCHWs has not yet reached all those who are eligible, and some have been upgraded pending receipt of training. Maternal health care in rural areas is usually provided by ANMs who have had at least 18 months training. There are relatively few staff nurse posts in rural areas, and these posts are often unfilled [11]. Table 1 presents levels of training of different nursing personnel in Nepal.

**Table 1:** **Nursing entry level qualifications and training**

**Course**	**Entry level**	**Length of training**
Maternal and Child Health Worker	Grade 8	3 months
Auxiliary Nurse Midwife	Grade 10	18 months
Proficiency Certificate Level (PCL) in Nursing (Staff Nurse)	Grade 10 (including Science, Maths and English)	3 years
Bachelors in Nursing (Generic BSc)	Grade 10 plus 2 in Science	4 years
Bachelors Nursing	PCL Nursing and 2 years work experience	2 years
Master of Nursing	BSc	2 years

Government guidelines for remote areas also recommend that additional staff be hired on short-term contracts at health facilities that either are upgrading to become birthing centres, are experiencing high absenteeism of staff, or have unfilled posts [12]. Little is known about the effect of this short-term employment (here onward referred to as ‘contracting’) on health workers, health service performance and provision [13]. We sought to measure the motivation of auxiliary nurse midwives (including MCHWs) and staff nurses in the context of these government recommendations, and therefore, we required locally valid measurement tools.

A review of the literature identified only one survey tool previously used to measure health worker motivation in a low-income setting. That tool was developed for use in district hospitals in Kenya [14] and subsequently tested in peripheral health facilities in Zambia [15]. It consists of 10 statements relating to three latent factors of motivation: organisational commitment, job satisfaction (including general motivation and burnout) and conscientiousness (including timeliness and attendance). Participants were asked to state the extent to which they agreed or disagreed with each statement. The tool was developed for use among health workers in a low-income setting, but it had only been used by the two studies cited above and had not been validated for use in South Asia. In this study, we explored the extent to which it is valid for use among ANMs and SNs in rural Nepal. We used qualitative and quantitative methods to assess the content validity (the extent to which the tool reflects all the aspects of a concept), the construct validity (the degree to which a tool measures what it claims to be measuring), the internal consistency (the degree to which several items that propose to measure the same general construct produce similar scores) and the reliability of the tool in this context [16].

## Methods

We used a reduced form of the COSMIN checklist to establish the local validity of the survey [17]. First, we evaluated content and construct validity by means of a literature review, interviews, focus group discussions and hypothesis testing. Then, we quantitatively tested for the internal consistency and reliability of the tool by calculating inter-item correlation and Cronbach’s alpha coefficients. Finally, we conducted a descriptive analysis to understand how motivation may be interpreted between different sub-groups.

Data collection tools were translated from English into Nepali by two trained female Nepali researchers who then collected and entered the quantitative and qualitative data. An additional two researchers translated recorded qualitative data. The tool was piloted with ANMs and SNs working in rural areas to check translation and comprehension. Participants gave written informed consent to participate, and the study was granted ethical approval from the Nepal Health Research Council.

We used data provided by a senior public health nurse in the Family Health Division of the Department of Health Services to purposively sample two districts with high numbers of contract nurses and one with low numbers of contract nurses. We sampled districts in the same geopolitical environment (Western Nepal) to enable comparability of data. One district with high levels of contracting was a remote hill district (Dailekh) with a Human Development Index (HDI) score of 0.422. The other two districts (Banke and Kailali) were in the plains. Banke had an HDI of 0.475, and Kailali had an HDI of 0.467 [18]. Our sampling was driven by practical and theoretical concerns. We used a mixed methods case study design, with the health facility as the case. We sought to compare health facilities with and without contract ANMs and SNs. The potential absence of nursing personnel, and lack of data at the district level about the numbers of nursing personnel on different types of contract, meant that we could not, *a priori*, design a sampling framework to ensure representation of the population. Therefore, we approached 16 health facilities per district, which had been conducting deliveries for the longest time, and aimed to interview all the nursing personnel in the health facility. Most study participants were ANMs (Table 2). There was variation between ANMs and SNs and their working contexts, which enabled comparative analysis to explore motivation theories. We collected data from health facility management committees (HFMCs), women’s groups and the in-charge at each health facility to explore triangulation and meet broader research objectives. Data were collected in sub-health posts (SHPs) (13), health posts (HPs) (29), primary health centres (PHCs) (10), hospitals (4) and district- and central-level stakeholders (12) from April to June 2013. Central-level stakeholders who deal with national-level nurse policy issues were purposively sampled and interviewed.

**Table 2:** **Demographic characteristics and motivation scores of nursing personnel**

**Variable**	***N***	**%**	**Mean motivation score**	**SD**
District				
Dailekh	40	29.2	4.74	0.31
Kailali	59	43.07	4.56	0.38
Banke	38	27.74	4.65	0.36
Age group				
18–19 years	6	4.38	4.56	0.37
20–29 years	60	43.8	4.53	0.39
30–39 years	43	31.39	4.68	0.32
40–49 years	25	18.25	4.83	0.23
≥50 years	3	2.19	4.70	0.38
Ethnicity				
Dalit Hill	4	2.92	4.80	0.30
Dalit Terai^a^	1	0.73	3.18	—
Disadvantaged Janajati Hill	14	10.22	4.69	0.28
Disadvantaged Janajati Terai	16	11.68	4.52	0.42
Relatively advantaged Janajati	9	6.57	4.71	0.33
Upper caste	93	67.88	4.65	0.33
Job title				
Upgraded MCHW	8	5.84	4.97	0.07
ANM	89	64.96	4.61	0.36
Senior ANM	23	16.79	4.73	0.21
Staff nurse	13	9.49	4.39	0.47
Senior staff nurse	3	2.19	4.91	0.09
Sister in-charge^a^	1	0.73	4.27	—
Type of current health facility				
Zonal hospital	13	9.49	4.64	0.37
District hospital	13	9.49	4.52	0.30
Primary health centre	24	17.52	4.59	0.41
Health post	64	46.72	4.63	0.36
Sub-health post	23	16.79	4.76	0.31
Time spent in current post				
Less than 1 month	12	8.76	4.51	0.50
1–6 months	23	16.79	4.55	0.30
7–12 months	6	4.38	4.65	0.21
1–2 years	22	16.06	4.69	0.31
≥2 years	74	54.01	4.66	0.37
Salary after tax (Nepali rupees)^b^				
Less than 5,000	5	3.65	4.15	0.69
5,000–9,999	39	28.47	4.56	0.38
10,000–14,999	71	51.82	4.69	0.28
15,000–19,999	21	15.33	4.69	0.35
More than 20,000^a^	1	0.73	—	—

^a^Indicates only one observation for this characteristic. As a result, the standard deviation is not calculated.

^b^96.29 NPR = 1 US dollars.

### Literature review

The 10 statements in the tool were reviewed against the literature on motivation. Our search strategy was focused on studies of motivation of health workers in low-income countries. We used PubMed and reviewed the reference lists of relevant articles. The aims of this process were 1) to test the assertion of Mbindyo et al. [14] that the statements explore the latent factors of motivation including organisational commitment, job satisfaction and conscientiousness and 2) to identify whether other latent factors exist that were not explored through the 10 statements. This process served as a first check of content validity.

### Qualitative data collection

Qualitative data were collected using semi-structured interviews and focus group discussions in one hill district (Dailekh) and one plains district (Banke). We were unable to collect qualitative and quantitative data from 7 in-charges and 51 nursing personnel because they were not at the health facility when we visited. One ANM in a hospital refused to be interviewed. We were unable to contact two HFMCs. Semi-structured interviews were conducted with SNs (13), ANMs (65) and health facility in-charges (25). In each district, semi-structured interviews with the public health nurse (PHN) and the district health officer (DHO) were also conducted. Focus group discussions (FGDs) were conducted with local women’s group members (31) and HFMCs (30) at each health facility. There were 6 to 15 people in an FGD, and discussion took up to 2 hours in duration. In nine health facilities in Banke, district women or HFMC participants spoke Abadhi or Hindi. Researchers used an ANM or health volunteer to translate where needed. JM observed a sample of quantitative and qualitative data collection in every district (22 ANMs and SNs, 1 PHN, 2 DHOs, 3 in-charges, 2 women’s group members, 3 HFMCs).

ANMs and SNs were asked to comment on their own motivation and the motivation of their work colleagues. We asked them to explain the reasons why they had described their colleagues or themselves as motivated or less motivated. Women’s group members, in-charges and HFMCs were asked about the motivation of specific ANMs and SNs and the reasons for this. We asked district- and central-level participants to tell us what type of ANMs and SNs were motivated, why and what motivates them.

Qualitative data were digitally recorded, and researchers translated qualitative data directly into English from the recordings. To check the quality of translation, a researcher back-translated three pages of four randomly selected interviews into Nepali and compared with the recordings. There were few discrepancies.

To explore content validity, JM coded data from all participants according to the domains represented in the tool and domains emergent from the data. To explore construct validity and internal consistency, JM sampled ANMs and SNs on the basis of their qualitative interviews. Twelve ANMs and SNs who appeared motivated and three ANMs or SNs who appeared particularly unmotivated were sampled. Data from ANMs and SNs was compared with data from women’s groups, HFMCs and health facility in-charges to triangulate findings. Lastly, ANM and SN motivation scores were examined to understand the extent to which they reflected the qualitative data and to check the reliability of the tool.

### Quantitative data collection: reliability and internal consistency

Quantitative survey data was collected in all three districts from 137 ANMs and SNs. Seventy-eight of these nursing personnel had also participated in qualitative data collection. Survey responses were recorded by researchers on paper forms and entered into a database at the site of data collection. There were no missing observations and all 137 records were used in the analysis. Quantitative data were analysed independently by NB using STATA version 13.

The motivation tool comprised 11 statements or ‘items’. Participants were asked whether they strongly agreed, agreed, disagreed or strongly disagreed with each item. The responses were coded to assign a higher value to those who were motivated and a lower value to those who were not.

We had no *a priori* reason to give higher weighting to any one item, and previous work in this area treated the items equally [14]. Therefore, the tool was calculated as a simple, equally weighted average.

The first test for internal consistency was conducted by calculating the degree to which the items in the tool were related to each other. If two items are highly correlated, then one of the two items will add little additional information about individual motivation. However, if items are uncorrelated, they measure different traits or dimensions of motivation. We calculated Spearman’s rank correlation coefficients for all 11 items to understand how they were related to each other.

We quantitatively measured the reliability of the tool by calculating Cronbach’s alpha (*α*) coefficient of internal consistency [19,20]. Cronbach’s alpha assesses the degree to which a set of items measures a single latent dimension. The *α* value for the motivation index was calculated and compared against acceptability thresholds [21-24].

After testing for internal consistency and reliability, we conducted exploratory descriptive analysis of the data. We first explored the distribution of motivation scores among ANMs and SNs. We then tabulated ANMs’ and SNs’ average motivation score by sociodemographic characteristics to see how their scores varied.

## Results

### Literature review: does the tool represent theoretical ‘best practice’?

The initial content validation through literature review identified one latent motivation factor: ‘self-efficacy’ not captured within the existing tool. This was identified as an important concept in the longer Mbindyo tool [15] and in several other publications [2-4,25-31]. We added a self-efficacy statement to the tool before it was translated into Nepali. In English, this statement reads as: “I am confident about my ability to do my job”.

### Qualitative results: how is motivation defined in western Nepal?

Generally, participants felt that ANMs and SNs who were doing their job were motivated: “*Everyone is motivated. They have all been doing their work*” (ANM HP). Some participants discussed job dissatisfaction among nursing personnel, and a sign of motivation was that they were working despite this unhappiness: “*they might feel demoralised and become inefficient in their work. Despite having these feelings they still have to do the work*” (ANM HP). When asking about the difference in motivation between nursing personnel, a few participants felt that ANMs and SNs who were receiving better terms and conditions were more motivated: “*The government nurses are more motivated. The services and facilities are good for government nurses*” (In-charge HP). Another in-charge in a health post told us: “*if it was possible to give nurses facilities (more leave, more pay, food allowances, pension) then their motivation towards their work would increase*.”

Most of the characteristics and actions used by participants to define and identify motivation related to conscientiousness: working hard, taking responsibility for work, being energetic, performing or striving towards high performance, being sincere or being determined: “*they seem to be doing work actively*” (ANM HP). Other related outcomes of motivation were confidence and a lack of carelessness. Careless, unmotivated ANMs and SNs delayed patient treatment unnecessarily, wasted time chatting, took unnecessary, long leave or did personal work during work hours: “*there is no-one that makes excuses to get out of work, like they are ill or they have personal work to do. It’s not like that here*” (ANM HP). Patient care, having good interactions with patients and not keeping them waiting were also emphasised: “*Staff are co-operative towards patients. The daily activities of the staff here really has a positive effect on patients*” (ANM hospital). Absenteeism was also a strong indication of lack of motivation: “*if staff didn’t come to the office daily, people wouldn’t have been able to get services. Because all the staff come, people have been getting good services, and staff are providing good care*” (ANM SHP). Participants did not mention timeliness as an indicator of motivation.

ANMs and SNs who were older or more senior and yet still working hard were exceptional and therefore motivated: *“they can manage cases even though they are old, so I think they are motivated and confident about their work”* (ANM hospital). But generally, younger ANMs and SNs were described as more motivated than older nursing personnel. A district-level stakeholder told us: “(older nurses) *say ‘I am over 50 years old now I don’t need to work hard anymore’. They look for excuses so that they don’t have to work.*” Another district-level stakeholder told us that this is because: “*(younger nurses) want to learn and they want to get more experience…older nurses say you ‘are a girl of 20 or 25 years old, you do the work and I will observe.’*” Many ANMs and SNs were keen to learn and develop their career within nursing, indicating an alignment of personal and organisational goals: “*I am very excited to see the new (abnormal) cases, I feel good about that…I have the opportunity to learn new things*” (ANM hospital).

Several central-level stakeholders emphasised that the job of ANMs and SNs was to care, and therefore, motivated nursing personnel should have an intrinsic ‘feeling’ of empathy, pride or a sense of higher purpose: “*if there is no feeling of serving people, then compassionate care will not come from their heart.*” Many ANMs and SNs felt a responsibility to provide care in their local area, and this motivated them: “*living here gives me the opportunity to develop our place and give services to the people of my area*.” (ANM PHC). ANMs and SNs were proud when women were happy with their services, and they were able to provide good-quality services: “*We need to counsel women about many things. I am proud to say that this health institution has been providing a good counselling service*” (ANM HP). Women also noticed this: “*we find the nurses motivated. They are always happy and pleased that they are working here.*”

The exertion of extra effort was also seen to define a motivated person and represented any action that went beyond normal expectations. For example, a willingness to transfer knowledge was indicative of motivation in a colleague, as it was not routinely expected: “*I think (other nurses) are motivated as they are more experienced than me. They even teach me the things that I don’t know*” (ANM HP). ANMs and SNs defined enduring personal hardship while working as a sign of motivation, for example being in remote places with few facilities or being away from their families: “*She is living alone without her family and providing 24 hour services, that is why I must say that she is motivated*” (SN PHC).

Many ANMs and SNs and other participants described motivation as working well together with other colleagues: “*they will never say that conducting deliveries is not our responsibility. They all work togethe*r” (women’s group). Coordination and flexibility is important when providing care in a remote place, and ANMs and SNs felt their colleagues were motivated when they exhibited these characteristics: “*While working, the person who has good relations with everyone is motivated and the one who can’t cooperate is not motivated*” (ANM hospital). Another ANM in a PHC told us: “*Everyone is motivated. Everybody helps each other.*” Although this may be more of a determinant of motivation, in our context it was also an outcome of motivation.

Our analysis shows that the construct validity of the tool is confirmed to a certain extent. All three latent concepts in the tool were also represented in the qualitative data: organisational commitment, job satisfaction and conscientiousness. A statement about absenteeism should replace the statement about timeliness, and indicators of exerting extra effort and being a teamworker should be added to the tool. Some data demonstrated a different understanding of motivation than the definition used in this study [3]. Some participants defined motivation as ‘just doing your job’, and others defined it in terms of seniority or salary paid: if you were paid more money, you were motivated. This indicates the importance of deconstructing concepts when designing tools.

### Quantitative results: internal consistency and reliability

This section uses survey data and accepted statistical tests to measure the internal consistency and reliability of the tool with nursing personnel in rural Nepal.

The survey was administered to 137 female ANMs and SNs. Most were from Kailali (43%), and the lowest proportions were from Banke (28%). Most were aged between 20 and 29 years (44%). Almost two thirds of the study population belonged to an upper caste group (Table 2), while approximately 25% belonged to Dalit or disadvantaged groups. The majority of the study participants were ANMs (65%), and most worked in health posts (47%). Very few ANMs and SNs worked in district or zonal hospitals (9.5%, respectively). More than half of ANMs and SNs had worked in their current health facility for more than 2 years (54%), and only 9% had worked in their current post for less than 1 month.

We calculated the inter-item correlation coefficients to quantitatively evaluate the internal consistency of the motivation tool. For the tool to be internally consistent, the items in the tool should be moderately correlated with each other [24,32]. Table 3 presents a matrix of the correlation coefficients. Overall, all items are positively and moderately correlated with each other. Some exceptions are the correlations between item 1 and item 2, and item 1 and item 5. Item 1 refers to timeliness. All ANMs and SNs reported being punctual to work.

**Table 3:** **Inter-item correlation matrix**

**Item**	**M1**	**M2**	**M3**	**M4**	**M5**	**M6**	**M7**	**M8**	**M9**	**M10**	**M11**
M1	1.0000										
M2	−0.0017	1.0000									
M3	0.0204	0.4184	1.0000								
M4	−0.0666	0.2367	0.2596	1.0000							
M5	0.0003	0.3579	0.5376	0.3095	1.0000						
M6	0.1468	0.1462	0.1568	0.3227	0.4001	1.0000					
M7	0.0861	0.2440	0.2811	0.2317	0.3954	0.3454	1.0000				
M8	−0.0172	0.3989	0.2515	0.4582	0.4345	0.4041	0.3833	1.0000			
M9	0.1599	0.3618	0.3279	0.3773	0.3005	0.2404	0.1404	0.4659	1.0000		
M10	0.0250	0.5241	0.3426	0.3594	0.4025	0.2339	0.4117	0.4686	0.5079	1.0000	
M11	0.1926	0.2315	0.3157	0.2918	0.2970	0.2339	0.0853	0.3448	0.4122	0.4117	1.0000

Note: M1 refers to item 1 in the motivation tool, M2 to item 2 and so on.

The *α* coefficient of internal consistency was used to measure the reliability of the motivation index constructed from the survey data. The calculated *α* value was 0.7918. This value was then compared to the acceptability threshold values presented in Table 4. The tool is considered to be internally consistent if *α* is equal to or bigger than 0.70. As the *α* value for the motivation tool was above the 0.7 threshold value, the tool can be considered internally consistent in this context.

**Table 4:** **Cronbach’s alpha (** ***α*** **) acceptability thresholds** [20]

**Cronbach’s alpha (** ***α*** **)**	**Internal consistency**
*α* ≥ 0.9	Excellent
0.8 ≤ *α* < 0.9	Good
0.7 ≤ *α* < 0.8	Acceptable
0.6 ≤ *α* < 0.7	Questionable
0.5 ≤ *α* < 0.6	Poor
*α* < 0.5	Unacceptable

The high response rates were an indication of the acceptability and comprehensibility of the motivation tool. The results from the internal consistency tests offer encouraging evidence of the reliability of the motivation tool, with both the correlation coefficients and the *α* value at acceptable levels.

**Figure 2:**
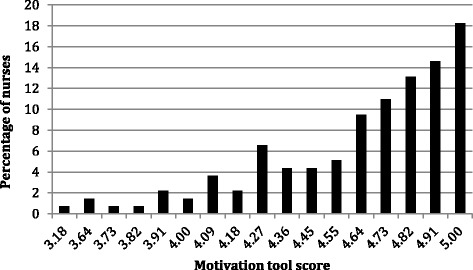
Distribution of motivation score among nurses.

We explored variations in the motivation score by sociodemographic and worker characteristics. The survey enables the calculation of a motivation score between 0 and 5, where a score of 0 denotes no motivation and 5 denotes the highest level of motivation. The mean motivation score in our sample was 4.63 (SD 0.36). The median motivation score was 4.7272 and the mode was 5. The distribution of the motivation score among the nursing personnel is presented in Figure 2. While there were no outliers, the distribution of motivation scores was slightly skewed to the right (skewness = −1.26).

We examined the mean motivation scores by demographic characteristics (Table 2). ANMs and SNs from Dailekh were more motivated (4.74, SD 0.31) than those who worked in the other two districts. Older ANMs and SNs in the 40−49-year age group were more motivated (4.83, SD 0.23) than those who were in other age groups. This is consistent with Mutale et al.’s findings [15]. In addition, nursing personnel belonging to the Dalit Hill ethnic group (4.8, SD 0.30) were more motivated than nursing personnel from other ethnic groups.

Upgraded MCHWs (4.97, SD 0.07) and senior staff nurses (4.91, SD 0.09) were more motivated than nursing personnel working in other posts. Again, this is consistent with Mutale et al.’s study, where senior nurses were more motivated that junior nurses. Those working in sub-health posts also had higher levels of motivation (4.76, SD 0.31). ANMs and SNs who had spent 7 months or more working at the current health facility were more motivated than others. Again, the finding that ANMs and SNs who spent more time in a health post were more motivated than those who had spent less time in a health post was consistent with that of Mutale et al. Finally, ANMs and SNs who earned a higher net income were more motivated. In particular, those earning between 10,000 and 14,999 Nepali rupees (104 and 155 US dollars) per month and between 15,000 and 19,999 Nepali rupees (155 and 208 US dollars) per month were highly motivated, with a mean motivation score of 4.69 (SD 0.28) and 4.69 (SD 0.35), respectively.

Analysis of mean motivation scores of ANMs and SNs with different demographic characteristics indicates that the motivation index could differentiate between high and low levels of motivation among ANMs and SNs. Furthermore, the motivation index was also able to capture variations in the motivation of ANMs and SNs who had different socioeconomic circumstances.

### Comparing qualitative and quantitative data: further insights into construct validity and reliability

To assess whether the tool accurately captured ANM and SN motivation, we compared semi-structured interview data from 15 nursing personnel, with their motivation scores. ANMs and SNs were purposively sampled from interview data, as they appeared ‘clearly motivated’ or ‘clearly unmotivated’. In addition, we considered data from HFMCs, women’s groups and in-charges (Table 5).

**Table 5:** **Comparing qualitative and quantitative motivation data**

**Auxiliary nurse midwife/staff nurse identification code**	**Motivation score (5 = highly motivated)**	**Qualitative interpretation**	**Auxiliary nurse midwife/staff nurse**	**HFMC, women’s group, in-charge**
1023	5	Not motivated	“I am from the local area so I cannot say no whether it is night or day or it is festival. Also sometimes I don’t feel appreciated.”	“She does the work but I think she is not interested in it, or she may not be interested in this occupation” (in-charge)
			“I am fed up with everybody (in management)…I don’ t have anyone who is in a high post…I know that everywhere opportunity is grabbed by the help of nepotism and favouritism so I know nobody does anything”	“She is always in hurry to leave the office at 2 pm after completing her work” (in-charge)
			“I cannot (go to training) as I have to give time for home. That’s why I don’t care about that”	
1009	5	Motivated	“Last year the volunteer and I were rewarded (by HFMC)…it feels really good to such respect in front of everyone”	“She really gives proper facility to all the women there. She loves the patients” (women’s group)
			“I always feel like wanting to go to training and learn things”	“She only goes to her house once in a year for the annual festival. She is the one who works the most” (in-charge)
			“During delivery cases people look for me to do the delivery…because of this admiration from the community and the connection with them it is very difficult for me to leave this place.”	“She really does work hard.” (HFMC)
1027	5	Motivated	“you enjoy and feel good when your work and reports are good”	“The other nurse does more work than nurse 1027” (in-charge)
			“I have a habit of doing work, I enjoy and talk with other staff”	“The other nurse is inspired to work” (HFMC)
2028	5	Motivated	“Women usually meet me before getting out of the health facility, so I like the fact that they want me to help them, and I feel about that”	“Their attitude towards the short term nurse is terrible…I suggested for rotation and asked for help (to prevent her from working 24 hours a day seven days a week)…and they didn’t speak and didn’t show up for duty.” (in-charge)
			“Whenever there is a case, the whole responsibility falls on your shoulders and won’t even feel like going to sleep until it is finished”	“The other nurse is working harder. She is working at night time as well as helps everywhere even during the day time” (HFMC)
1036	5	Motivated	“Everyone here is positive towards me and everyone wants me to work over here, everyone asks me not to leave this place”	“She goes everywhere. She never says no if we ask her to do something” (in-charge)
				“The nurses seem motivated, they are eager to work. They are also doing a good job. They are doing work with interest” (women’s group)
1037	5	Motivated	“I have been able to provide services like I wanted. People come for the delivery after calling us or informing us, so we counsel them and do the deliveries.”	”Both nurses are good, its not good to compare.” (HFMC)
			“I believe I am in the best team ever….everyone is supportive here.”	“The attendance of the nurses is equal. They come regularly” (HFMC)
1028	4.91	Motivated	“Whenever the patients arrive here, they immediately come asking for me. Even if I am in the middle of my meal, I rush here. I am really curious towards the patients and provide services with immense care and love.”	“Nurse 1028 has lots of work to do. (The other health worker) signs her attendance…and then (she leaves)…Her home is far away. Nurse 1028 has more work to do” (women’s group)
			“I have been providing services from deep within my heart and soul.”	“Nurse 1028 stays in the health institution any time, whenever the patient comes. She doesn’t even know when day and night has passed.” (HFMC)
			“I like doing everything here, like checking up ANC, checking the condition of delivery, and doing deliveries. There is nothing that I don’t enjoy here.”	
2035	4.9	Motivated	“It’s easier to work here, its not difficult working here…all the staff seem co-operative.”	“The nurses behave well, they speak politely, and they also handle the cases properly” (women’s group)
				“The nurses are always happy and pleased to be working here” (women’s group)
2034	4.82	Motivated	“I feel happy because after I came here the situation improved. I did all the work, including out patients department, because no staff used to come here.”	“We see nurse 2034 all the time in the health post whether it is night or day. We like that” (women’s group)
			“(The other staff) give all the work to me. They are skilled, but they don’t want to do the work.”	“Nurse 2034 has won the hearts of the local people” (in-charge)
				“Nurse 2034 works very hard. She even sweeps the floor.” (HFMC)
2003	4.8	Motivated	“Some fulfil their duty very well, but some do it only to a minimum level… contracted nurses (like me) do well…and permanent nurses…are a bit careless.”	“One who has come more recently (like nurse 2003) respects the senior nurses, so there is some difference in work.” (in-charge)
				“Every person has a different nature. Some behave very well and speak politely but some are very strict and speak rudely.” (women’s group)
2036	4.7	Motivated	“During the night there isn’t anyone to help. I have to do everything. Its difficult.”	“There is one nurse (2036) who has been here for a long time, so she is the one whom we are involved with so even while check up with her we feel very comfortable and we find her behaviour very well. The way she treats and speaks to us is good.” (women’s group)
				“I haven’t been able to see any differences (between nurses)…they are all satisfied.” (in-charge)
1011	4.7	Motivated	“I am excited to gain more practical knowledge”	“The nursing staff are good. They are from a new generation and they are behaving well because they are trained” (women’s group)
				“Permanent nurses attend a lot less than the contracted nurses (like 1011)” (in-charge)
2020	4.27	Motivated	“Women talk about all their problems with me…everyone knows me. They all respect me.”	“While I come here I see both the nurses” (women’s group)
			“Staff teach me things I don’t know, and …they praise me for my work.”	“The behaviour of the nurses is good” (women’s group)
				“They are both motivated, as far as I can tell.” (in-charge)
1005	4.18	Less motivated	“The work is split between us, but I have to work harder than the other nurse”	“There are no differences in the work (between the nurses)…The (other) permanent nurses are more motivated…their grade is increasing (for the years that they work). The services and facilities for government staff are good.” (in-charge)
			“I feel that people here take advantage of (me). They think we should work 24 hours a day, and there are delays in getting our salary…”	
			“The possibility of getting a job elsewhere is low. I must give services after office time, and I feel I am working effectively.”	“When nurse 1005 was not recruited, there was no service. It is good now.” (HFMC)
2006	3.18	Less motivated	“I cannot apply whatever I learned while studying to be a staff nurse…the community do not allow us to work according to our system.”	“In relation to the work, it has been really good. The nurses have done their work at 12 midnight, and in the morning also. So they are really sincere in their work.” (in-charge)
			“If there is post partum haemorrhage and the woman died, the people here won’t understand that it was because they didn’t allow me to work, and they will come to hit us.”	“(Nurses) are okay.” (HFMC)

Quantitative self-scoring by ANMs and SNs and interview data about how they felt appear to be fairly consistent, apart from participant 1023 and participant 2020. Participant 1023 scored very highly, but her interview and others’ perceptions of her indicated she was less motivated. Participant 2020 had a comparatively low score, but qualitative data indicated she was motivated. Triangulation between ANM and SN scores and others’ observations of their behaviour were less consistent. Participant 2028 and participant 2006 felt differently from how others perceived them. Although older nursing personnel had higher quantitative motivation scores, this was not always supported by the qualitative data from other participants.

## Discussion

Our research with ANMs and SNs in rural Nepal sought to explore the validity, internal consistency and reliability of an established survey tool developed to measure health worker motivation in Kenya. We have already discussed the validity, internal consistency and reliability of the survey, and therefore in this section, we focus on how the survey could be adapted to improve its appropriateness in the Nepali context. Where appropriate, we also compare our findings to those of Mbindyo et al. [14].

Motivation, as defined by Franco et al. [3], was captured to a large extent by the tool. Qualitative and quantitative analysis showed that item 1 on the scale had weaker validity. All ANMs and SNs said that they were punctual when coming to work, and timeliness was not emphasised in the qualitative data. Therefore, this tool item did not help to differentiate levels of motivation. However, in Mbindyo et al.’s study, timeliness was one of the constructs that drove variation in motivation scores. We recommend that the first question on the scale be replaced with a question about attendance or absenteeism when it is used in our context. Qualitative data revealed that exertion of extra effort and teamwork were important components of motivation. These were also other constructs that drove variation in motivation scores in Mbindyo et al.’s study but were not adequately represented in the 11-item tool. These concepts have been measured in other tools measuring the determinants of health worker motivation, but in our study, they were also outcomes of motivation [27,33].

The discrepancy between high quantitative motivation scores of older nursing personnel and qualitative data that younger nursing personnel often work harder emphasises the need for qualitative data to understand these differences. Qualitative data was also useful in revealing the presumption of high motivation among those with better terms and conditions, when we found evidence that often ANMs and SNs with worse terms and conditions were working harder. Therefore, we recommend conducting qualitative research, with a smaller purposive sample, along with the motivation tool where possible.

Most participants had high motivation scores with means of 4.63. This may be because the tool only uses questions that were positively worded. Positive wording may bias participants to rank themselves as more motivated. We recommend changing this tool to have a mix of positively and negatively worded statements to help deal with this bias.

The fact that most ANMs and SNs scored highly on the motivation tool also suggests a potential risk in using this tool to measure change over time or after the implementation of an intervention. If scores are already high, how can they go higher? Qualitative data (not presented here) suggest that there are many factors that demotivate ANMs and SNs in this context, but they were not always reflected in their quantitative scores. This may suggest that the survey is not sensitive to smaller differences in motivation. This study did not set out to assess the sensitivity of the survey, and this is noted both as a limitation of this study and an area for further work.

### Limitations

While we were able to suggest some changes to improve the validity of the tool in this context, this study has a number of limitations. Firstly, qualitative findings suggest that absenteeism was an indicator of lack of motivation and yet we only conducted the quantitative validation survey among nursing personnel who were present in the health facility at the time of data collection. This may explain the high mean motivation scores observed, which may reflect a positive bias in the quantitative data collected. Secondly, our understanding of who is ‘unmotivated’ in this context may be limited by the fact that when asked directly, nursing personnel and other participants were reluctant to state that a health worker was less motivated. This may be exacerbated by the fact that a few in-charges, women’s group members and HFMCs were not well informed about the ANMs and SNs in their health institution (particularly those who were not in regular attendance). This may also have introduced a positive bias. Thirdly, the study included only female nursing personnel. Therefore, the extent to which these findings would also apply to other male health workers is uncertain. Finally, we were unable to test for the criterion validity of the tool, as there is no ‘gold standard’ tool for the measurement of motivation against which we could compare the calculated motivation scores. We did not re-administer the tool at a second time point, as we did not want to place additional burden on the participants. Therefore, we were unable to test the responsiveness of the tool. Finally, high mean rates observed in the quantitative data may be a result of the positive biases described above, or they may indicate a lack of sensitivity in the tool. This is highlighted as an area for further research.

## Conclusion

This study used three sources of data to validate an available survey tool designed to measure health worker motivation. Literature review, primary qualitative and quantitative data were used to explore the content validity, construct validity, internal consistency and reliability of the tool in Nepal. We found that with minor modifications, the tool provides valid and internally consistent measures of motivation among nursing personnel in this context.

The qualitative data suggest that, in this context, motivation is revealed through action. A conscientious approach to work including regular attendance, active participation and high levels of patient care are all seen as signals of high motivation. Conversely, low motivation is indicated by absenteeism, a lack of active engagement in the workplace and poor patient care. The qualitative findings suggest that adding statements about exertion of extra effort and teamwork, and changing the punctuality statement would improve the tool in this context. Quantitative analysis demonstrates that this tool is valid for use among ANMs and SNs and perhaps other health workers in the Nepalese context. We recommend that statements in the tool should be phrased both positively and negatively to help prevent bias. We also recommend the collection of qualitative data to help understand motivation tool scores. To our knowledge, this is the first study validating a survey tool for measuring health worker motivation in this context, and we believe that it constitutes an important start for further work in this area.

## Abbreviations

ANM: Auxiliary nurse midwife
DHO: District health officer
HDI: Human Development Index
HP: Health post
MCHW: Maternal child health worker
PHN: Public health nurse
PHC: Primary health centre
SBA: Skilled birth attendant
SHP: Sub-health post
SN: Staff nurse
SD: Standard deviation
